# Augmented reality for orthopedic and maxillofacial oncological surgery: a systematic review focusing on both clinical and technical aspects

**DOI:** 10.3389/fbioe.2023.1276338

**Published:** 2023-11-22

**Authors:** Naqash Nasir, Laura Cercenelli, Achille Tarsitano, Emanuela Marcelli

**Affiliations:** ^1^ Oral and Maxillo-Facial Surgery Unit, IRCCS Azienda Ospedaliero-Universitaria di Bologna, Bologna, Italy; ^2^ Orthopedics and Traumatology Department, IRCCS Azienda Ospedaliero-Universitaria di Bologna, Bologna, Italy; ^3^ eDIMES Lab-Laboratory of Bioengineering, Department of Medical and Surgical Sciences, University of Bologna, Bologna, Italy; ^4^ Maxillofacial Surgery Unit, Department of Biomedical and Neuromotor Science, University of Bologna, Bologna, Italy

**Keywords:** augmented reality, mixed reality, orthopedics oncology, maxillofacial oncology, surgery, tracking

## Abstract

This systematic review offers an overview on clinical and technical aspects of augmented reality (AR) applications in orthopedic and maxillofacial oncological surgery. The review also provides a summary of the included articles with objectives and major findings for both specialties. The search was conducted on PubMed/Medline and Scopus databases and returned on 31 May 2023. All articles of the last 10 years found by keywords augmented reality, mixed reality, maxillofacial oncology and orthopedic oncology were considered in this study. For orthopedic oncology, a total of 93 articles were found and only 9 articles were selected following the defined inclusion criteria. These articles were subclassified further based on study type, AR display type, registration/tracking modality and involved anatomical region. Similarly, out of 958 articles on maxillofacial oncology, 27 articles were selected for this review and categorized further in the same manner. The main outcomes reported for both specialties are related to registration error (i.e., how the virtual objects displayed in AR appear in the wrong position relative to the real environment) and surgical accuracy (i.e., resection error) obtained under AR navigation. However, meta-analysis on these outcomes was not possible due to data heterogenicity. Despite having certain limitations related to the still immature technology, we believe that AR is a viable tool to be used in oncological surgeries of orthopedic and maxillofacial field, especially if it is integrated with an external navigation system to improve accuracy. It is emphasized further to conduct more research and pre-clinical testing before the wide adoption of AR in clinical settings.

## 1 Introduction

Augmented reality (AR) is a technology that allows the fusion of digital content into the real environment. The achieved augmented continuum is a virtual world in which virtual objects are overlaid on real elements, in the surrounding actual environment (Azuma et al., 2001).

The first AR system using a Head Mounted Display (HMD) was developed by Sutherland in 1968 (Feiner, 2002). Since its discovery, AR technology has been utilized by experts in many areas; such as entertainment, sports, gaming, retail, and also medicine. Indeed, the recent technological advancements in headsets and computer hardware resulted in many companies, especially in the entertainment sector, investing in AR devices which have become increasingly available and accessible. Therefore employed also in health-related applications particularly in the surgical fields.

When applied to surgery, the AR allows to improve the user’s perceptual and comprehensive ability by projecting three-dimensional underlying anatomy directly onto the user’s retina (via HMDs) or on a display screen.

AR in surgery has enormous potential to help the surgeon in identifying tumor locations, delineating the planned dissection planes, and reducing the risk of injury to invisible structures. Therefore, using AR in the operating room (OR) could be helpful in performing surgical tasks in a more accurate way. HMDs are particularly beneficial for AR surgical applications since they intrinsically provide the surgeon with an egocentric viewpoint, and offer improved ergonomics if compared to traditional computer-assisted surgical systems. This allows surgeons to concentrate on the task at hand without having to turn their heads away from the surgical field to constantly look at imaging monitor. The most ambitious goal in surgery is to use AR for intraoperative navigation. This involves taking data from preoperative imaging and using anatomical anchors in the operating field to register the two representations in real time.

Registration is an important step in computer-assisted surgical navigation in order to correlate the virtual content and the real surgical scene. In this context, the registration error can be defined as; the measurement of how much the virtual objects displayed in AR appear incorrectly positioned relative to the real environment.

For virtual-to-real surgical scene registration, AR systems typically use a camera coupled to a device marker; such as QR code, anchored to the patient (marker-based registration). Another option is marker-less registration which includes a combination of location data (from Global Positioning System), inertial measurement unit (IMU) data, and computer vision to track image features such as scene depth, the object surface, and object edges (Venkatesan et al., 2021).

The core of the registration modality is tracking, which means to determine and follow the position and orientation of an object with respect to some reference coordinate system over time.

Over the past decade, with the advent of multimodal and high-detailed 4D medical imaging (Bradley, 2008), numerous surgical specialties have integrated AR into their surgical workflow, namely; neurosurgery (Cannizzaro et al., 2022), urological surgery (Bianchi et al., 2021; Schiavina et al., 2021; Roberts et al., 2022), ophthalmology (Li et al., 2021), gastrointestinal endoscopy (Mahmud et al., 2015), cardiovascular surgery (Rad et al., 2022), spinal surgery (Molina et al., 2021a), breast surgery (Gouveia et al., 2021), and thyroid surgery (Lee et al., 2020). Some authors utilized AR to perform lateral skull-based surgery for cerebellopontine angle tumor (Schwam et al., 2021) and some used it in open hepatic surgery (Golse et al., 2021). Moreover, AR has also been employed in procedures such as perforator flap transfer (Jiang et al., 2020) and percutaneous nephrolithotomy (Ferraguti et al., 2022).

Orthopedic and Maxillofacial surgeries have been pioneers in the use of AR in a surgical setting (Barcali et al., 2022).

These two surgeries may represent very promising fields for the future clinical implementation of AR, since they are based on bony hard tissues which make it easier to have fixed references, i.e., bony structures, to be used for ensuring an accurate virtual-to-real scene registration between preoperative (virtual) and intraoperative (real) views.

Regarding orthopedic surgery, Alexander et al. (2020) formulated a 3D augmented reality system for the placement of acetabular component during total hip arthroplasty (THA) and found it to be more precise and faster than standard fluoroscopic guidance. Similarly, Ogawa et al. (2018) found AR to be more accurate when comparing it to conventional goniometer for acetabular cup placement during THA. In 2019, Tsukada et al. (2019) conducted an *in vitro* study on sawbone models for employing AR during total knee arthroplasty and concluded that the system provided accurate measurements for tibial bone resection. Consequently, in 2021, the same authors, formulated prospective cohort study on 72 patients. They emphasized that AR-assisted navigation to resect distal femur is more precise than the conventional method (Tsukada et al., 2021).

Augmented reality and its tools have emerged as a new paradigm also in spinal surgeries. Many authors have validated the use of AR navigation for the precise placement of pedicle screw (Elmi-Terander et al., 2018; Elmi-Terander et al., 2019; Gibby et al., 2019; Dennler et al., 2020) and some compared its accuracy with free-hand approach (Elmi-Terander et al., 2020). In 2021, Molina et al. (2021b) conducted the first human trial of using an FDA approved AR-HMD (X-vision Spine System, Augmedics) and demonstrated its clinical and technical accuracy in spine surgery.

In the context of oral and cranio-maxillofacial surgery, AR applications are of increasing interest and adoption (Badiali et al., 2020).

Sharma et al. (2021) proposed a marker-less AR navigation system algorithm with greater precision and faster processing time for jaw surgery. Similar to the article on marker-less image registration for jaw experiments published by Wang et al. (2019), this study demonstrated its clinical viability through minimal registration error and processing time.

Some other experiences of marker-less AR navigation have been reported for assisting the harvesting of periosteum pedicle flap and osteomyocutaneous fibular flap in head and neck reconstruction (Battaglia et al., 2020), as well as for guiding osteotomies in pediatric cranio-facial surgery (Ruggiero et al., 2023).

Similarly, recent studies in dental implantology have demonstrated the efficacy of AR for displaying dynamic navigation systems (Pellegrino et al., 2019; Shrestha et al., 2021). Ma et al. (2019) proposed an AR-assisted navigation with cone beam computed tomography (CBCT) registration method to attain the desired dental implant precision. They compared the navigation method to physician’s experience and concluded that AR guidance had better outcomes in terms of mean target error and mean angle error. Budhathoki et al. (2020) emphasized the use of AR navigation to visualize deep-seated anatomy, narrow areas and to provide positioning of surgical instruments to avoid positioning error complications during jaw surgery. Moreover, Gao et al. (2019) employed AR in mandibular split osteotomy. Same as, Pietruski et al. (2019) who incorporated AR navigation and cutting guides for mandibular osteotomies in 2019 and concluded that this technology can enhance the surgeon’s perception and hand-eye coordination during mandibular resection and reconstruction procedures.

Although AR technology has a long history in orthopedic and maxillofacial surgery, a complete analysis of its clinical and technical application on oncological cases is still lacking.

In this literature review, we provide the comprehensive up-to-date overview on current clinical applications of AR in orthopedic oncology and maxillofacial oncology, pointing out its benefits and current limitations. Moreover, we also elucidate the different technological aspects of AR used in each of these experiences to give an insight on how AR can be administered in oncological surgical scenarios.

## 2 Materials and methods

### 2.1 Searching criteria

This systematic literature review was conducted on PubMed/Medline and Scopus databases using the terms, “Augmented Reality” AND “Orthopedic oncology,” “Mixed Reality” AND “Orthopedic oncology,” “Augmented Reality” AND “Maxillofacial” AND “oncology,” “Mixed Reality” AND “Maxillofacial” AND “oncology” and “Augmented Reality” AND “Head and Neck” AND “cancer.” The same search was also attempted using the term “Cranio-maxillofacial” AND “oncology” in the place of “Maxillofacial” AND “oncology,” from which, however, no additional results were obtained with respect to what was already found. Searches for both specialty domains were done separately and 2 independent users performed search until 31 May 2023. Relevant articles of only last 10 years were included in this review paper. Manual search was also done in references of papers to see missing of any relevant paper. PRISMA-guidelines were kept in mind while preparing this review article.

The SPIDER (Sample, Phenomena of Interest, Design, Evaluation, Research type) method was used to construct the suitable research question: “Can augmented reality be considered a beneficial tool in orthopedic and maxillofacial oncological fields in achieving surgical accuracy?”

This review addresses this question by focusing on both clinical and technical aspects of AR in these two surgical disciplines, as well as on reporting current limitations and benefits.

Due to the qualitative and mixed-method nature of the included articles and the heterogeneity of the data, the term “evaluation” was left intentionally broad.

We performed the study selection based on the following inclusion criteria: studies on augmented reality applications in either orthopedic or maxillofacial field, only focused on oncology; studies reporting applications on different targets (e.g., phantoms, cadavers, animals and patients). All articles with either quantifiable or qualitative outcomes on augmented reality confined to both specialties with case reports were included.

Exclusion criteria were the following: articles “not relevant” (i.e., not related to augmented reality, not strictly related to oncological surgery, not related to orthopedic or maxillofacial surgery); articles with language of publication other than English; theses; conference papers; editorials; book chapters; review articles (as Review articles typically do not include sufficient specifics regarding the recommended solutions and are also considered as secondary source, therefore they cannot be used in data extraction process).

### 2.2 Data extraction and analysis

All the search articles available till 31 May 2023 were screened by title first and then abstract.

The authors, date of publication, study design, and data from the eligible articles were tabulated in Microsoft Excel^®^ (Microsoft Corporation, WA). All articles meeting the inclusion criteria were read carefully and stratified following two parallel perspectives: a clinical one, i.e., focusing on the specific surgical application, the type of study (on phantoms, on cadavers, on animals, on humans), the anatomical region of interest, the virtual information provided to surgeon, and a technical one, i.e., the registration/tracking modality the type of AR display, the achieved registration and surgical accuracy).

Key findings of each article were also stated in given tables in the Results section for both orthopedic and maxillofacial specialties, and also depicted in bar histograms. However, meta-analysis could not be performed due to heterogeneity of literature. All these findings were validated by a second independent investigator to ensure the correct data acquisition and selection of the appropriate relevant literature.

Due to the fact that the included study designs exhibited a significant level of variability, as is often observed in the case of new technologies, they are developed individually with distinct features. Consequently, conventional approaches for evaluating the risk of bias were not suitable for use in this context. The authors generally evaluated and assessed the risk of bias to be low or negligible for data description, but it could be high for the analysis of the effectiveness of approaches used in these studies. Furthermore, none of the articles included in the review refers to a specific methodological protocol.

## 3 Results

The initial search of the PubMed/Medline and Scopus databases was completed on 31 May 2023, and all available articles were scrutinized using the above-described criteria.

In the following paragraphs, an analytic overview of the selected papers and their classification were presented for both orthopedic oncology and maxillofacial oncology.

**FIGURE 1:**
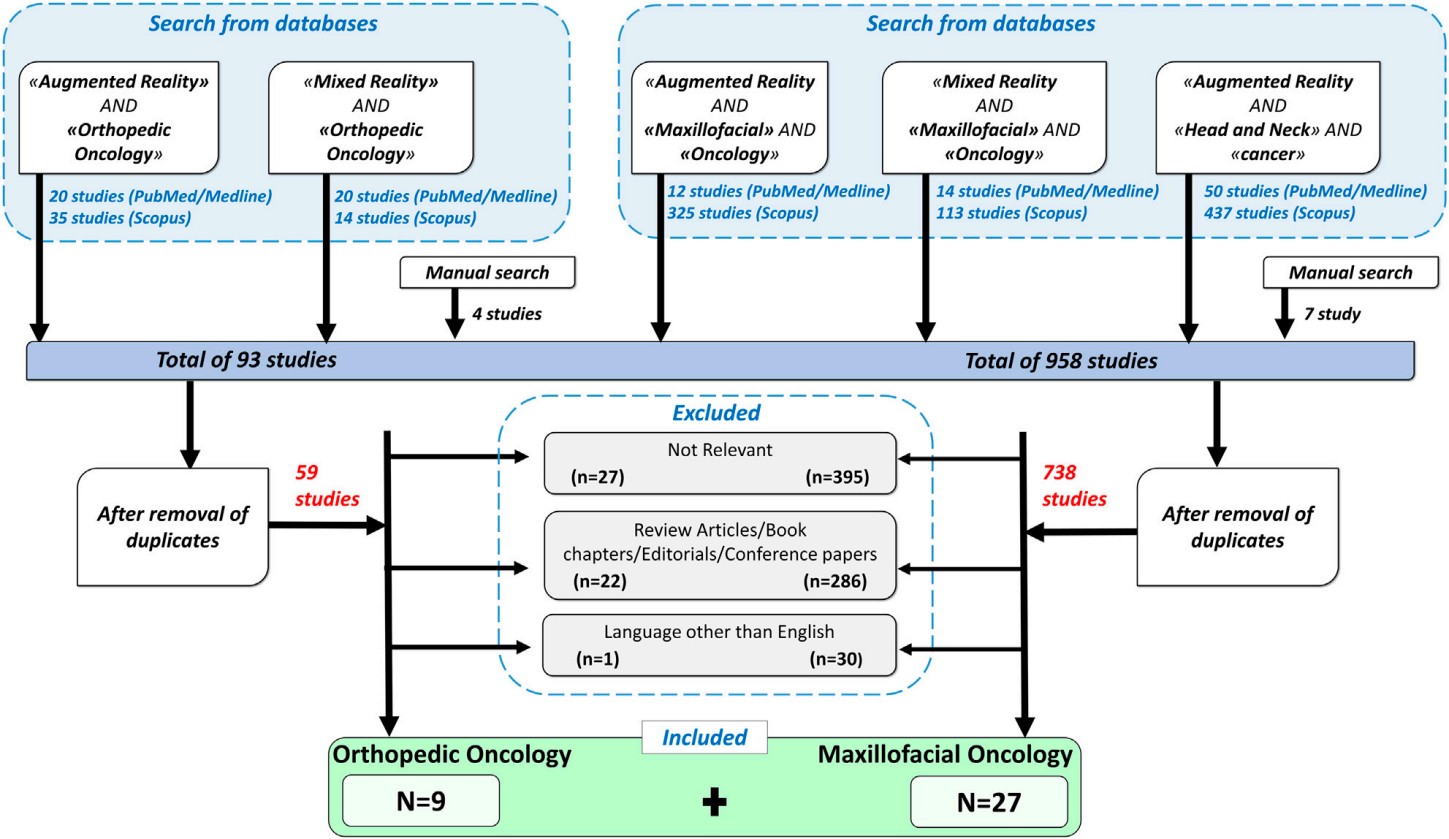
A flowchart showing inclusion and exclusion criteria used for the search, and the resulting selected papers.

As depicted in flowchart (Figure 1), the databases search for orthopedic oncology returned 89 results while manual search yielded 4 publications. Fifty-nine articles remained after removing duplicates based on titles and abstracts. According to the inclusion criteria, only 9 of the 59 articles were included in this review. Other articles were excluded for the following reasons: “not-relevant” (*n* = 27), review articles/book chapters/editorials/conference papers (*n* = 22), non-English articles (*n* = 1).

Similarly, for CMF oncology, 951 articles were found through PubMed/Medline and Scopus search and 7 from manual search. Out of total 958, 738 articles remained after removal of duplicates. Based on above defined criteria, 27 publications were included and others were eliminated for the following reasons: “not-relevant” (*n* = 395), review articles/book chapters/editorials/conference papers (*n* = 286), non-English language (*n* = 30).

For the included articles, both clinical and technical aspects are summarized separately in given tables for both specialties (Tables 1–4). For the clinical aspects, we classified the papers according to: the specific surgical application, the number of cases involved, the involved anatomical region, the type of study (i.e., on phantom, on cadaver, on patient), the virtual information provided to augment the surgeon’s view (Tables 1, 3). For the technical aspects, we considered the type of AR display and device, the used registration/tracking modality, as well as the achieved registration error and surgical accuracy (measured in mm) (Tables 2, 4). For each included article we provided in a separate table a brief description of the study and the major outcomes (Tables 5, 6).

**TABLE 1:** Distribution of studies by clinical aspects of AR in Orthopedic Oncology.

Reference	Authors	Year	Target of study	Virtual content	Specific surgical application	Anatomical region	No. of cases
Moreta-Martinez et al. (2021)	Moreta et al.	2021	Phantom and Patient	Bones, tumors, cutting planes	2 myxofibrosarcoma,1 liposarcoma, 1 Ewings sarcoma, 1 fibrous dysplasia, 1 undifferentiated pleomorphic sarcoma	Femur, thigh, calf, pelvis, shoulder	6
Cho et al. (2017)	Choi et al.	2017	Animal cadaver	Coloured template of tumor with normal bone and safety margin	Simulated femur tumor	Femur	Total = 123 AR = 82 con = 41
Cho et al. (2018)	Choi et al.	2018	Animal cadaver	Tumor, surgical plane and safety margin	Simulated pelvic tumor	Pelvis	Total = 36 AR = 18, con = 18
Choi et al. (2017)	Choi et al.	2017	Animal cadaver	Tumor, safety margin, resection and saw plane	Simulated pelvic tumor	Pelvis	Total = 60, AR = 30, con = 30
Molina et al. (2021c)	Molina et al.	2021	Patient	Tumor and osteotomy trajectory	L1 chondroma	Spine	1
Abdel Al et al. (2020)	Abdel et al.	2020	Patient	MRI images with tumor	Soft tissue sarcoma	Foot	1
García-Sevilla et al. (2021a)	Garcia-Sevilla et al.	2021	Phantom	Bones and PSI	Simulated Pelvic tumor	Pelvis	6
Moreta-Martinez et al. (2018)	Moreta et al.	2018	Phantom and Patient	Skin, bone and tumor	Ewings sarcoma	Tibia-fibula	1
Pose-Díez-de-la-Lastra et al. (2022)	Pose-Díez-de-la-Lastra et al.	2022	Phantom and Patient	Bone, tumor and surgical guide	Extraosseous Ewing’s sarcoma and Undifferentiated pleomorphic sarcoma	Tibia-fibula, shoulder	2

**TABLE 2:** Distribution of studies by technical aspects of AR in orthopedic oncology.

Reference	Authors	Year	AR display type	AR device	Registration/Tracking modality	No. of cases	AR registration error	AR surgical accuracy
Moreta-Martinez et al. (2021)	Moreta et al.	2021	Screen-based	Smartphone iphone 6	Marker-based (cubic marker)	6	Mean registration error: 2.80 ± 0.98 mm	n.a
Cho et al. (2017)	Choi et al.	2017	Screen-based	Tablet (Surface Pro 3)	Marker-based (ArUCo code)	Total = 123 AR = 82 conv = 41	n.a	Mean resection error (difference between the obtained and the planned surgical margin): AR-assisted resection: 1.71 ± 0.25 mm; Conventional resection (manual measurement): 2.64 ± 0.5 mm
Cho et al. (2018)	Choi et al.	2018	Screen-based	Tablet (Surface Pro 3)	Marker-based (ArUCo code)	Total = 36 AR = 18 conv = 18	n.a	Mean resection error (difference between the obtained and the planned surgical margin):AR-assisted resection: 1.59 ± 4.13 mm; Conventional resection (based on CT image): 4.55 ± 9.7 mm
Choi et al. (2017)	Choi et al.	2017	Screen-based	Tablet (Surface Pro 3)	Marker-based (ArUCo code)	Total = 60 AR = 30 conv = 30	Mean registration error: 0.58 ± 0.22 mm	Mean resection error (i.e., difference between the obtained and the planned surgical margin): AR-assisted resection: 0.15 ± 1.02 mm; Conventional resection (manual measurement): 2.89 ± 4.30 mm
Molina et al. (2021c)	Molina et al.	2021	HMD	X-Vision Augmedics	Marker-based	1	n.a	n.a
Abdel Al et al. (2020)	Abdel el at	2020	screen-based	Smartphone (Samsung Galaxy A5)	Marker-less	1	n.a	n.a
García-Sevilla et al. (2021a)	Garcia-Sevilla et al.	2021	Screen-based/HMD	Smartphone/HoloLens 2	Marker-based	6	n.a.	Median MOD (Maximum Osteotomy Deviation) between planned and real osteotomy planes (for realistic phantom): AR-assisted positioning of surgical guide (smartphone): 1.54 mm; AR-assisted positioning of surgical guide (HoloLens2): 1.84 mm; Freehand positioning: 3.37 mm
Moreta-Martinez et al. (2018)	Moreta et al.	2018	HMD	HoloLens 1	Marker-based	1	(Phantom): Registration error (Root Mean Square Error (RMSE) in AR point localization): 2.90 mm (Clinical): n.a.	n.a.
Pose-Díez-de-la-Lastra et al. (2022)	Pose-Díez-de-la-Lastra et al.	2022	HMD	HoloLens 1 and 2	Marker-based	2	Registration error (RMSE in AR point localization): −2.16 mm (HoloLens2–Leg phantom); −2.83 mm (HoloLens1–Leg phantom); −3.11 mm (HoloLens2–shoulder phantom);	n.a.

**TABLE 3:** Distribution of studies by clinical aspects of AR in maxillofacial oncology.

Reference	Authors	Year	Target of study	Virtual content	Specific surgical application	Anatomical region	No. of cases
Chan H et al. (2022)	Chan et al.	2022	Phantom	Tumors and cutting planes	Simulated Maxillary tumors	Maxilla	5
Sahovaler et al. (2021)	Sahovaler et al.	2021	Phantom	Tumors and cutting planes	Simulated Sinonasal tumors	Sinonasal	4
Shi et al. (2022)	Jiafeng shi et al.	2022	Phantom	Mandible and osteotomy line	Mandibular tumor	Mandible	1
Ceccariglia et al. (2022)	Ceccariglia et al.	2021	Patient	Skin, tumor with surrounding normal bone and cutting planes	1 maxillary squamous cell carcinoma,1 osteomyelitis of left jaw, 1 osteomyelitis of left mandible	Maxilla, mandible	3
Ochandiano et al. (2021)	Ochandiano et al.	2022	Phantom and Patient	Mandible with teeth, implant position and angulation, splint (tracker)	8 mandibular, 1 maxilla,1 tongue and 1 hard palate	Mandible, maxilla, tongue, hard palate	7
Gsaxner et al. (2019)	Gsaxner et al.	2019	Phantom and Patient	Bone and tumor mass	Head and neck cancers (not specified)	Head and neck (not specified)	8
Pepe et al. (2019)	Pepe et al.	2019	Phantom	Tumor, reference markers, slice of PET-CT scan	Simulated Head and neck tumor	Head and neck (not specified)	1
García-Sevilla et al. (2021b)	García-Sevilla et al.	2022	Phantom and Patient	Bone, tumor, surgical resection margin, splint for tracking	Adenoid cystic carcinoma	Hard palate	1
Tel et al. (2021)	Tel et al.	2021	Phantom and Patient	Skull with maxillary sinuses, muscles and fat, tumor, infraorbital nerve, optic nerve	Inferior orbital compartment tumors (3 cavernous hemangioma, 1 neurofibroma, 1 schwannoma)	Orbit	5
Gsaxner et al. (2021)	Gsaxner et al.	2021	Phantom	Skull and tumor, orthogonal slices of imaging in anatomical planes	Head and neck carcinoma (not specified)	Head and neck (not specified)	1
Sugahara et al. (2021)	Sugahara et al.	2021	Patient	Tumor, nasal cavity and maxillary sinus with surrounding normal skull	Maxillary calcifying odontogenic cyst	Maxilla	1
Shaofeng et al. (2023)	Shaofeng Liu et al.	2023	Phantom and Animal	Mandible, teeth, resection lines, surgical saw, fixation screw	Bilateral mandibular simulated tumor	Mandible	9 model trials, 12 animal trials
Prasad et al. (2023)	Prasad BA et al.	2023	Human Cadaver	Simulated cancer	20 different simulated Head and neck cancers	Head and neck (not specified)	20
Gao et al. (2022)	Gao et al.	2022	Phantom	Skull and virtual recontouring plan	Craniofacial fibrous dysplasia	Maxilla	5
Kim et al. (2020)	Jin Kim et al.	2020	Patient	Skull, tumor and cutting planes	Malignant fibrous histiocytoma of the maxilla	Maxilla	1
Yang et al. (2022)	Yang et al.	2022	Patient	Mandible with tumor and surgical planes	Mandibular tumor	Mandible	4
Tang et al. (2022)	Tang et al.	2022	Patient	Skull, tumor, surgical planes, probe	Maxillary and mandibular tumor	Maxilla, mandible	7
Scolozzi and Bijlenga (2017)	Scolozzi et al.	2017	Patient	Skull and tumor	Pleomorphic adenoma of lacrimal gland	Orbit	1
Cercenelli et al. (2022)	Cercenelli et al.	2022	Phantom	Bone, vessels, skin, osteotomy line	Skin paddle harvesting in osteomyocutaneous fibular flap	Mandible	1
Scherl et al. (2021)	Scherl et al.	2021	Patient	Surface of the face, mandible, masseter, parotid gland, tumor	Parotid tumor	Parotid Gland	6
Necker et al. (2023)	Necker et al.	2022	Human Cadaver	Mandible, tumor	Mandibular tumor	Mandible	1
Han et al. (2022)	Lin et al.	2022	Phantom and Patient	Arteries, veins, bone tissues	Mandibular ameloblastoma	Mandible	1
Zhao et al. (2023)	Zhao et al.	2022	Cadaver	Fibula, osteotomy lines	Mandible (application: fibular flap harvesting)	Mandible	7
Modabber et al. (2022)	Modabber et al.	2021	Cadaver	Iliac crest with planned osteotomies	Mandibular tumor (application: iliac crest harvesting)	Mandible	10
Meng et al. (2021)	Meng et al.	2021	Cadaver	Fibula, fibular flap with cutting planes	Mandible (application: fibula flap harvesting)	Mandible	1
Battaglia et al. (2019)	Battaglia et al.	2021	Patient	Skin, fibula, cutting guides, arteries	Mandible (application: fibular flap harvesting)	Mandible	3
Winnand et al. (2022)	Winnand et al.	2022	Phantom	Iliac crest with planned osteotomies	Mandible (application: iliac crest graft harvesting)	Mandible	10

**TABLE 4:** Distribution of studies by technical aspects of AR in maxillofacial oncology.

Reference	Authors	Year	AR display type	AR device	Registration/Tracking modality	No. of cases	AR registration error	AR surgical accuracy
Chan H et al. (2022)	Chan et al.	2022	Projector-based	Portable high-definition projector (PicoPro, Celluon Inc.)	Marker-based	5	Fiducial registration error of ≤1 mm	AR-guided osteotomies had high similarity with the preplanned, with interclass correlation index (ICC) close to 1 (0.893) in “adequate” (5–15 mm) margins
Sahovaler et al. (2021)	Sahovaler et al.	2021	Projector-based	Portable high-definition projector (PicoPro, Celluon Inc.)	Marker-based	4	Fiducial registration error of ≤1 mm	n.a.
Shi et al. (2022)	Jiafeng shi et al.	2022	HMD	HiAR G200 AR Glasses	Marker-less	1	Surface matching error: 0.64 ± 0.28 mm	Mean surgical resection error: 0.49 ± 0.37 mm
Ceccariglia et al. (2022)	Ceccariglia et al.	2021	HMD	HoloLens 2	Marker-less	3	n.a.	Surgical resection error (deviation between AR-projected osteotomy and the one planned and performed with cutting guide: <2 mm
Ochandiano et al. (2021)	Ochandiano et al.	2022	Screen -based	Smartphone (iphone 6)	Marker-based	7	n.a.	n.a.
Gsaxner et al. (2019)	Gsaxner et al.	2019	HMD	HoloLens 1	Marker-less	8	Target registration error: 9.2 ± 1.5 mm	n.a.
Pepe et al. (2019)	Pepe et al.	2019	HMD	HoloLens 1	Marker-less Marker-based	1	Mean registration error along (x, y, z): Marker-less: (3.3 ± 2.3, −4.5 ± 2.9, −9.3 ± 6.1) mm; Marker-based (7.0 ± 2.1, −12.5 ± 2.5, −19.0 ± 2.0) mm	n.a.
García-Sevilla et al. (2021b)	García-Sevilla et al.	2022	Screen based	Smartphone (Iphone 6)	Marker-based	1	n.a.	Median surgical resection error: 0.40 mm
Tel et al. (2021)	Tel et al.	2021	Screen based	Smartphone (Iphone 12 Pro)	Marker-less	5	n.a.	n.a.
Gsaxner et al. (2021)	Gsaxner et al.	2021	HMD/PC	HoloLens 1/PC	Marker-less	1	Registration error between a few millimeters of up to 2 cm	n.a.
Sugahara et al. (2021)	Sugahara et al.	2021	HMD	HoloLens 1	Marker-based (attach to splint)	1	n.a.	n.a.
Shaofeng et al. (2023)	Shaofeng Liu et al.	2023	HMD	HiAR G200 AR glasses	Marker-less Marker-based	9 model trials, 12 animal trials		Model Trials Distance deviations: 1.62 ± 0.38 mm (mandible) 1.86 ± 0.43 mm (fibula); 1.67 ± 0.70 mm (fixation screws) Angular deviations: 3.68 ± 0.71° (mandible); 5.48 ± 2.06° (fibula); 7.50 ± 1.39° (fixation screws) Animal trials Distance deviations: 0.93 ± 0.63 mm (condyle); 2.01 ± 2.49 mm (mandibular angle); 1.41 ± 0.61 mm (mandible); 0.88 ± 0.22 mm (tibiofibular bones) Angular deviations: 6.81 ± 2.21° (mandible); 6.47 ± 3.03° (tibiofibular bones)
Prasad et al. (2023)	Prasad BA et al.	2023	HMD	HoloLens 2	Manual	20	Average relocation error of 4 mm ± 3.9 mm	n.a.
Gao et al. (2022)	Gao et al.	2022	HMD	HoloLens 1	Marker-based	5	n.a.	Mean surgical error: 1.036 ± 0.081 mm
Kim et al. (2020)	Jin Kim et al.	2020	Screen-based/HMD	Self-developed AR viewer (Vive pro, monitor)	Marker-based	1	n.a.	Mean surgical error: 2.77 ± 1.29 mm
Yang et al. (2022)	Yang et al.	2022	HMD	HoloLens 1	Marker-less	4	Registration error <1 mm	Maximum surgical error: 6.08 mm
Tang et al. (2022)	Tang et al.	2022	HMD	HoloLens 2	Marker-less	7	n.a.	Mean surgical error: 1.68 ± 0.92 mm
Scolozzi and Bijlenga (2017)	Scolozzi et al.	2017	Screen-based	Microscope-based AR system	Marker-less	1	n.a.	n.a.
Cercenelli et al. (2022)	Cercenelli et al.	2022	Screen-based HMD	Tablet HoloLens 2	Marker-less	1	Registration errors ranging between 1–5 mm	Accuracy: 2.0 mm (100% success rate with HoloLens; 97% with tablet)
Scherl et al. (2021)	Scherl et al.	2021	HMD	HoloLens 1	Manual	6	Mean error of the alignment: 1.3 cm	n.a.
Necker et al. (2023)	Necker et al.	2022	HMD	HoloLens 2	Manual	1	n.a.	n.a.
Han et al. (2022)	Lin et al.	2022	HMD	HoloLens 2	Manual	1	n.a	n.a
Zhao et al. (2023)	Zhao et al.	2022	Screen-based	Monitor	Marker-based	7	n.a	Length difference: 1.18 ± 0.84 mm, Angular deviation: 5.45 ± 1.47°, Volume overlap rate: 95.31% ± 2.09%, Average surface distance: 1.22 ± 0.12 mm.
Modabber et al. (2022)	Modabber et al.	2021	Projector-based	ML750ST, Optoma projector	Marker-less	10	n.a	Angulation of the osteotomy plane: AR group: 14.99 ± 11.69° Cutting guides group: 8.49 ± 5.42° Osteotomy plane distance: AR group: 2.65 ± 3.32 mm Cutting guides: 1.47 ± 1.36 mm
Meng et al. (2021)	Meng et al	2021	HMD	HoloLens 1	Manual	1	n.a	Mean location of the fibular osteotomies: 2.11 ± 1.31 mm Angular deviation of the fibular segments: 2.85° ± 1.97° Intergonial angle distances: 7.24 ± 3.42 mm
Battaglia et al. (2019)	Battaglia et al.	2019	Screen-based	Tablet	Marker-less	3	n.a	n.a
Winnand et al. (2022)	Winnand et al.	2021	Projector-based	ML750ST, Optoma projector	Marker-less	10	n.a	Average discrepancy in osteotomy plane angulation AR: 10.21 ± 7.22° Cutting guides: 6.98 ± 4.70 Mean variations between the osteotomy planes and the planned trajectories: AR: 2.29 ± 1.98 mm Cutting guide: 1.32 ± 1.00 mm

**TABLE 5:** Brief description of each article with aim and major outcomes, on Orthopedic Oncology.

Reference	Author, date	Brief description with aim and outcomes
Moreta-Martinez et al. (2021)	Moreta et al., 2021	Aim: Authors introduced a surgical workflow to orthopedic oncology by combining 3D printing and a smartphone-based AR application. A 3D-printed reference marker was used for virtual-to-real patient registration. The system was experienced on six patient-specific phantoms and in two clinical cases. This system was evaluated in terms of visualization accuracy and usability during the whole surgical workflow Results/Conclusion: Phantom experiments provided a visualization accuracy (i.e., registration error) below 3 mm. Positive feedback was obtained from surgeons and patients
Cho et al. (2017)	Choi et al., 2017	Aim: Authors conducted an experiment on pig femurs using a tablet-based AR navigation system to evaluate the accuracy of AR-assisted oncological surgeries compared to conventionally performed surgeries. To simulate a bone tumor in the pig femur, a cortical window was made in the diaphysis and bone cement was inserted. A total of 164 surgical removals using the AR technique and 82 resections using the conventional procedure (i.e., manual measurement as per routine clinical practice) were performed Results/Conclusion: The mean resection error (i.e., the difference between the obtained and the planned surgical margin) was 1.71 mm (0–6) and 2.64 mm (0–11) for AR-based and conventional interventions, respectively (*p* < 0.05). In AR-based navigation resection, 90.2% of the times, the surgical margin of 10 mm was successfully attained, compared to only 70.7% in conventionally performed surgeries. The authors concluded that the accuracy of tumor resection achieved with the proposed AR-based navigation was satisfactory
Cho et al. (2018)	Choi et al., 2018	Aim: Authors conducted animal trials showing that AR navigation may prove useful in pelvic tumor resections. Researchers injected bone cement into the acetabular dome of 36 cadaver pig pelvises to compare the AR navigation technique and the conventional navigation technique (based on CT image). Each technique was assigned with 18 tumor pelvises to operate on with a safety margin of 1 cm Results/Conclusion: With marker-based AR technology, the mean margin of resection was 1.59 ± 4.13 mm, whereas in the conventional navigation it was 4.55 ± 9.7 mm. 100% AR-assisted resections had errors <6 mm as compared to 78% resections done using conventional method, showing the attainability of planned margins using AR. The authors further emphasized that more *in vivo* trials are needed before adopting it for clinical trials
Choi et al. (2017)	Choi et al., 2017	Aim: This study was aimed at achieving the margin of cancer resection in 60 porcine acetabular regions using AR navigation method and conventional method based on manual measurement (30 pelvises were assigned for each technique). A tablet PC was used for AR visualization and cuboidal reference markers were used for tracking. 3D dilation technique was used in simulated tumor model, and separation of this 3D dilation and actual tumor was deemed as safety margin. The target margin of resection was set at 10 mm. The surgical resection plane, determined by this safety margin, was adopted in accordance with the direction of cutting saw in real time, and minimum distance between the cutting saw and resection plane was measured Results/Conclusion: The mean fiducial registration error for anatomical landmarks was 0.58 ± 0.22 mm. After resection, analysis showed the measured resection margin to be 9.85 ± 1.02 mm for AR navigational resection as compared to 7.11 ± 4.30 mm with conventional resection. The reduction in standard deviation from 4.30 to 1.02 mm demonstrates the high precision of surgical resection margins using the AR method. With the conventional method, 1/4 of surgical procedures resulted in invasion of the 5 mm tolerance margin, while none of the surgeries using the AR method resulted in such invasion. Hence, AR navigation is proved to be more accurate for guiding surgical resection of complex bone tumors
Molina et al. (2021c)	Molina et al., 2021	Aim: Authors presented a first-ever case report using AR-assisted spinal surgery on L1 chondroma to perform *en bloc* wide osteotomy via posterior approach Clinical symptoms: A 69-year-old man experienced lower back pain and paresthesis 6 months prior to surgery. He had a history of lymphoma for which he received chemotherapy and radiation.He has been symptom-free since 2010 Investigations: Imaging by MRI revealed an interosseous lesion with spinal extension at L1 level. And chondroma was diagnosed through a biopsy Results/Conclusion: The minimal invasive *en bloc* procedure was performed with precise lumbar osteotomy and screw placement using AR-HMD devices and flippable tracker to avoid any line-of-sight obliteration. Postoperatively, there were no reports of long-term complications, and pathology confirmed a negative resection margin. Therefore, AR guidance assisted in achieving the intended navigational trajectories for the placement of pedicle screws
Abdel Al et al. (2020)	Abdel el at, 2020	Aim: Authors published a case report of a 39-year-old male with a deep-seated impalpable soft tissue (synovial fluid) sarcoma of the medial aspect of the left foot. The resection was done using smartphone-based AR guided MRI images to outline sarcoma superficially Clinical Symptoms: Two years ago, the patient complained of pain on the medial side of the left sole without a relevant medical history. This non-radiating pain intensified while walking, and topical analgesics had no effect Investigations: Imaging and biopsy were performed to detect the mass, and synovial sarcoma was diagnosed. The subsequent physical examination was unremarkable, and MRI revealed a bilobed lesion on the medial aspect of the left foot. In addition, the PET-CT scan revealed a small, mildly hypermetabolic lesion, but no distant metastases Results/Conclusion: A patient-specific AR application was devised to superimpose MRI images on foot landmarks. The tumors borders were outlined on skin using sagittal MRI images. Using AR-guided pre-operative markings for tumor localization, *en bloc* excision was performed complication-free, except for decreased sensation in the medial aspect of the foot. Furthermore, follow-up imaging ruled out any infection, recurrence, and metastasis. It was emphasized that this smartphone-based AR application is suitable for small and fixed tumor
García-Sevilla et al. (2021a)	Garcia-Sevilla et al. 2021	Aim: Authors conducted a pre-clinical study demonstrating the importance of AR to guide patient-specific instruments (PSI) placement in pelvic tumor resection. Six different ilium tumor scenarios were simulated for evaluation. In addition, six pairs of PSI were designed, resulting in two PSI’s for each case scenario. Experiment was devised using a smartphone and HoloLens 2 to test the system’s accuracy and compared it to freehand method. Two varieties of phantoms were utilized for assessment. i.e., conventional plastic pelvic bone (no silicone layer) and realistic phantom (silicone layer). System accuracy was analyzed based on PSI transformation from its intended position to its actual one, which provided the maximum osteotomy deviation (MOD) Results/Conclusion: The median values for MOD with smartphone or HoloLens 2 are found to be less than 2 mm in silicone phantom and below 1 mm in conventional plastic bone, whereas the median values for PSI placed with freehand were 3.37 mm for realistic phantom and 1.70 mm for non-silicone one. There was significant difference between freehand method and using either smartphone or HoloLens (*p* < 0.001). For phantom’s comparison, high errors were observed in all cases with silicone phantom (*p* < 0.001). In the end, authors found encouraging evidence that AR has the ability to overcome the current constraints of PSIs in a straightforward and efficient manner
Moreta-Martinez et al. (2018)	Moreta et al., 2018	Aim: Authors demonstrated an AR approach for Extra-osseous Ewing sarcoma (EES) of distal limb. Using patient-specific tools with marker attached, pre-surgical simulation was done on patient-specific phantom replicating Ewing’s sarcoma before testing during actual surgical intervention. Two sets of 3D models were printed. One was used for validation purposes (including conical holes in design) and the other for surgical application. For system precision, evaluation was done on the basis of surgical guide placement error and error in AR point localization for visualization. For that purpose, conical holes on both phantoms and surgical guide were used as a reference point for registration and error measurement. The measurement was accomplished using an optical tracking system (Polaris) Result/Conclusion: Surgical guide placement error and AR visualization error was measured in average root-mean square and valued 1.87 and 2.90 mm, respectively among all 3 repetition attempts. In addition, the complete workflow was implemented by an expert surgeon on actual surgical field using HoloLens, and satisfactory alignment was achieved. The authors believe that the developed pre-surgical system will pave the way for the creation of user-friendly AR systems that can be used in the medical industry for training, simulation, and guidance
Pose-Díez-de-la-Lastra et al. (2022)	Pose-Díez-de-la-Lastra et al., 2022	Aim: In this study, researchers evaluated the accuracy of HoloLens 1 and HoloLens 2 using two orthopedic oncological cases. For this purpose, they acquired patient-based phantom simulating EES of distal leg. Secondly, they examined the accuracy of HoloLens 2 on more complex case of right shoulder undifferentiated pleomorphic sarcoma. And finally, researchers employed HoloLens 2 on a real patient with a pleomorphic sarcoma of the right shoulder in operating room to evaluate its technical and ergonomic aspects. Additionally, patient-specific surgical guides were also designed to accommodate AR registration markers. Using an optical tracking system, the AR projection error was analysed by documenting the positions of AR spheres on a phantom surface. The procedure was repeated three times by three researchers Results/Conclusion: Leg phantom (EES) showed greater accuracy with HoloLens 2 with RMSE of 2.16 mm as compared to HoloLens 1 (RMSE = 2.833 mm). Significant difference among devices was noted (*p* < 0.05). In a case of pleomorphic sarcoma of the shoulder, the RMSE for HoloLens 2 was 3.108 mm. The increased error by HoloLens 2 in second case was due to larger size and dimensions of phantom. Lastly, HoloLens 2 were tested on actual patient with pleomorphic sarcoma and the surgeon praised the enhanced ergonomics of this HMD. In addition, a survey was conducted to compare both head gears and the HoloLens 1 received a score of 2.84 out of 5 as compared to 4 for the HoloLens 2 indicating the superior ergonomics of this device. In conclusion, HoloLens 2 had better results in terms of both accuracy and ergonomics when tested on orthopedic oncological cases

**TABLE 6:** Brief description of each article with aim and major outcomes, on Maxillofacial Oncology.

Reference	Author, date	Brief description with aim and outcomes
Chan H et al. (2022)	Chan et al., 2022	Aim: Authors conducted a preclinical study on phantoms to address the issue of margin control using AR. 5 phantom models with maxillary tumors were created and 5 resident surgeons carried out both AR-guided simulated resection and virtual unguided resections for comparison. 115 osteotomies were performed virtually and comparison was done on basis of intralesional cuts (≤0 mm), close (>0 mm and ≤5 mm), adequate (>5 mm and ≤15 mm) and exceeding distance (>15 mm) from tumor Results/Conclusion: Registration error was less than 1 mm for AR application. In context of surgical accuracy, Intratumor margin was 0% in AR-guided resection versus 1.9% in unguided simulation. Close margin also showed low percentage of 0.8% and 7.9% in AR-guided and unguided resections, respectively. In both cases, *p*-value was <0.0001. With a *p*-value of 0.018, the percentage was greater for the AR-guided simulation at 25.3% as opposed to 18.6% in the unguided simulation for “adequate” resection. No differences were noted for excessive margins. In addition, AR-guided simulations scored higher in mental demand, performance, effort, and frustration. According to authors, AR-guided resections had more similarities with pre-planned surgical planes. Consequently, they concluded that AR methodology improves negative margin through more precise rendering of preplan cutting planes
Sahovaler et al. (2021)	Sahovaler et al., 2021	Aim: Researchers conducted a preclinical study to compare approach of AR and intraoperative navigation (IN) on sinonasal malignancies removal. five surgeons performed simulations of virtual cuts to compare AR approach and advance IN approach on four tumor models. Unguided, AR, IN and AR+IN simulations were performed and statistically compared. Making intratumor cuts the key outcome, the others “close, adequate, and excessive distances” from tumor were also analyzed in percentages. Additionally, screening timing was calculated based on the information from gaze tracker headset Results/Conclusion: AR application registration error was <1 mm. Out of 335 cuts, percentage of intratumoral cuts were 20.7%, 9.4%, 1.2% and 0% for the unguided, AR, IN, and AR+IN simulations, respectively (*p* < 0.0001) showing the advantage of AR over unguided simulation. IN approach decreases intratumoral cuts as compared to AR alone approach. Whereas, combination of both AR and IN did not improve intratumoral rate significantly (*p*-value 0.5). The screening timing in unguided, AR, IN, and AR+IN turned out to be 55.5%, 0%, 78.5%, and 61.8%, respectively (*p* < 0.001). The screening time and workload score (NASA-TLX questionnaire Score) in AR+IN approach improves as compared to IN alone approach. Hence, the authors concluded that AR navigation improves open sinonasal tumors resections as well as overcome the attention-deteriorating-screening problem of IN. However, more works needs to be done on this application before clinical implementation
Shi et al. (2022)	Jiafeng shi et al., 2022	Aim: In this article, authors conducted a study based on marker-less tracking on mandibular edge for resection of benign maxillofacial tumor to avoid the complications caused by guiding plate. Before surgery, they replicated the 3D model for diseased bone for pre-surgical simulation and access the lines of resection of tumor Results/Conclusion: They analyzed the marker-less surface registration error and turned out to be 0.6453 ± 0.2826 mm (<1 mm), affected by system error and impact was ignored surgically. Surgical error was assessed using an experimental AR system and found out to be 0.4858 ± 0.3712 mm (<1 mm). The authors concluded that AR-guided marker-less navigation can effectively display and guide the surgical path. Therefore, it helps in achieving the desired results and has a positive impact on doctors
Ceccariglia et al. (2022)	Ceccariglia et al., 2021	Aim: In this study authors demonstrated the application of marker-less AR registration for removal of maxillofacial tumors, and performed the resectional surgeries on three patients suffering from oral tumors using AR. Two males and one female patients with the mean age of 56 years underwent seven group of osteotomies in total. These osteotomies were analyzed by comparing corticotomy lines drawn by AR guidance and customized cutting guides Results/Conclusion: The difference of under 2 mm was noted between AR projected osteotomy and customized cutting guide osteotomy, hence showing that marker-free AR navigation is achievable. However, the authors also emphasized that further research is needed to be done on marker-less facial registration for maxillofacial tumors resection despite being considered safe
Ochandiano et al. (2021)	Ochandiano et al., 2022	Aim: Authors published an article emphasizing the role of 3D printing, virtual surgical planning (VSP) and augmented reality in head and neck tumors ablation and dental implants. They included 11 patients. Out of which, 8 suffered from mandibular, 1 tongue, 1 maxilla and 1 hard palate carcinoma. Total of 56 implants were inserted, but 6 of them were withdrawn from data analysis due to unavoidable intra operative complications. Using intraoperative infrared optical navigation (for the first four patients), surgeons virtually planned and transferred the prosthetically driven dental implant placement to the patient. Finally, they used a combination of conventional static teeth supported 3D-printed acrylic guide stent, intraoperative dynamic navigation, and AR for final intraoperative verification for other seven patients. Differences in implants coronal, apical, and angular location between preoperative 3D planning and guided intraoperative placement were quantified Results/Conclusion: Due to jig registration instability, initial simple infrared navigated cases achieved low accuracy. Dynamic navigation cases that follow achieved 1–1.5 mm insertion point deviation with the help of highly stable acrylic static guides used as reference and to register markers. Hence, Image-guided surgery, 3D printing, and AR technology could be used to precisely plan, implantation and reconstructive surgeries. The authors went on to stress the importance of pre-clinical education in managing the technology’s steep learning curve
Gsaxner et al. (2019)	Gsaxner et al., 2019	Aim: Authors employed marker-free inside-out image to face registration for augmented reality in head and neck oncological cases. For that, they 3D printed eight phantom heads of subjects with tumors in this region and subsequently tested the application’s viability on human subject. For accuracy estimation, target registration error (TRE) was measured through five different landmarks and compared it with optical tracking system Results/Conclusion: TRE was repeated 10 times on each phantom and human subject reporting a mean TRE of 9.2 ± 1.5 mm, in comparison to error of high-precision optical tracking system of 3.9 ± 1.8 mm in translation and 4.9 ± 2.4° in rotation. Even though authors did not achieve the desired precision required for this type of intervention due to certain restrictions, they still believe that AR is a viable tool for these interventions
Pepe et al. (2019)	Pepe et al., 2019	Aim: In this article, authors employed a marker-less approach for a head and neck tumor demonstration on a 3D PET CT scan-acquired model. In model, *x* and *y* axes were marked along the horizontal and vertical axis of the tumor mass for evaluation purposes, whereas the z-axis depicts the direction of gaze. Red points were added to facial landmarks to measure the registration error against the green virtual points from the AR application, and an experienced user repeated the measurements four times. Automatic registration error was analyzed with and without user calibration Results/Conclusion: Errors with user calibration (markers) along the y, x, and z-axes were to be −4.5 ± 2.9 mm, 3.3 ± 2.3 mm and −9.3 ± 6.1 mm, respectively. These errors had nearly doubled values without user calibration (without markers) along these axes (−12.5 ± 2.5 mm, 7.0 ± 2.1 mm and − 19.0 ± 2.0 mm), indicating that user calibration guarantees a more precise registration. However, the most anticipated difficulty during this study appears to be physician’s limited depth perception, which restricted him from viewing the 3D model from certain angles. Moreover, the standard ISO-9241/110 feasibility questionnaire based on 5-point Likert Scale was conducted and overall feedback turned out to be positive
García-Sevilla et al. (2021b)	García-Sevilla et al., 2022	Aim: In this study, authors compared three different navigation techniques for *en bloc* resection of exophytic invasive adenoid cystic carcinoma of the hard palate. Preoperative simulation and accuracy of these surgical navigations based on pre-defined surgical margins were estimated on phantom and each simulation was repeated three times Results/Conclusion: Initially, an optical tracking system (OTS) facilitating registration using screws (five screws were attached to maxilla before image acquisition) was examined for surgical guidance. The median deviation from the desired surgical margin was 0.57 mm, but with lowest variations (IQR of 0.24 mm). Whereas, OTS enabling registration with surgical guide (splint), had median and IQR both higher than screw registration. AR navigation, on the other hand, had the lowest median of 0.40 mm, but with high variation exhibiting IQR of 0.89 mm. However, no statistical difference was noted in terms of accuracy (errors below 1 mm). But, owing to low error variations, OTS with screw registration was considered for real surgical intervention, giving the fiducial point registration errors of 0.77, 0.93 and 0.81 mm over three different repetitions. Additionally, AR was also tested in OR for qualitative study. Authors concluded that these navigational approaches are accurate and convenient for minimal invasive and conservational approaches
Tel et al. (2021)	Tel et al., 2021	Aim: The authors conducted a qualitative investigation into the use of computer-assisted pre-operative surgical planning, virtual endoscopy, and AR navigation for the resection of various histopathological masses in the inferior orbital compartment via transantral approach. Five patients with disease-related main signs and symptoms were included. Pre-surgical simulation was conducted on patient-specific 3D-printed model Results/Conclusion: Ultimately, surgery was performed under transmaxillary navigation guidance using virtual models to verify the correct surgical positioning throughout all essential stages of endoscopic transantral approach. Excisions were performed through a transantral approach on all patients without intraoperative or long-term postoperative complications. It was determined that each step of virtual planning can be replicated with relative precision during actual surgery. In the context of AR implementation, accurate tracking and overlaying were performed on each patient-specific phantom for preoperative simulation, highlighting its role during preoperative study, training, and actual surgery
Gsaxner et al. (2021)	Gsaxner et al., 2021	Aim: This article emphasized on practicability and utility of a designed AR system for head and neck tumor simulation and clinical settings. Eleven healthcare personnel with extensive experience in the head and neck region were recruited. During the initial segment of training, participants received a comprehensive introduction and demonstration of the HoloLens system using a patient phantom. The researchers were then requested to evaluate the AR system on a healthy volunteer with a simulated tumor in the head and neck region to demonstrate a clinical case during the testing phase Results/Conclusion: Registration error was noted between a few millimeters to 2 cm. After completing both phases (training and demonstration), physicians participated in system feasibility questionnaires and an informal interview. The AR usability evaluation received a System Usability score (SUS) of 74.8 ± 15.9 (>68 indicates above average) with a 5-point Likert scale of 4.5 ± 0.7 out of 5 (with “1” representing extremely negative and “5” representing extremely positive). According to users, however, registration precision and the time required for auto-registration were pressing concerns. In conclusion, clinicians find this AR system simple to learn and use, which improves their decision-making skills
Sugahara et al. (2021)	Sugahara et al., 2021	Aim: Authors presented a case report on the resection of maxillary calcifying odontogenic cyst and reconstruction using mixed reality Clinical Symptoms: 27 years old female had a gingival swelling in 2010 and left untreated due to the abstinence of discomfort. Later in 2016, during routine dental care, an x-ray revealed radiolucent area but again disregarded against the medical advice. Next year, upon displacement of left anterior maxillary teeth, patient was referred to our department Investigations: Physical examination revealed left nasal ala deformity along with intraoral and left upper lip swelling. The Electric pulp test showed vital reaction in this region but no pain upon pressure Pre-requisites: Partial resection of left maxilla from the lateral incisor to the second pre-molar with iliac cancellous bone and marrow grafting was planned. Virtual planning and estimation of graft quantity to fill post-resection cavity were accomplished prior to surgery. 3D model with all details on tumor and its resection was printed. During surgery, tumor and surrounding anatomical structures were virtually visualized by three surgeons using HoloLens Results/Conclusion: The resection was successfully executed according to plan and post-surgical void was reconstructed using iliac cancellous bone graft. At the 18 months follow-up, there were no complications or relapse. In conclusion, the incorporation of mixed reality during pre-surgical and intra-operative phases allows for accurate and secure surgery
Shaofeng et al. (2023)	Shaofeng Liu et al., 2023	Aim: Authors tested the feasibility and accuracy of marker-less contour-based AR registration for simulated mandibular tumor and fibula-based reconstruction on phantom models and subsequently compared this AR based system accuracy with surgical guides in animal trials. Virtual tumor tissue of right and left mandible was designed, and 9 mandibular and fibular models were 3D printed. Based on the trigger detection algorithm, virtual-real registration of osteotomy instruments for tracking and calibration of instrument’s angle was also accomplished. After virtual-real scene registration of instrument and lesion, mandibular resection, screw fixation and fibular reconstruction were accomplished under AR guidance and post-surgical CBCT data was analyzed for deviations from pre-operative planning Results/Conclusion: For model trials, the distance and angular deviation for mandibular osteotomy surface were 1.62 ± 0.38 mm and 3.68 ± 0.71°, respectively. And for fixation screws, it was 1.67 ± 0.70 mm and 7.50 ± 1.39°, respectively. In contrast, the distance and angular deviation of reconstructed fibular osteotomy were 1.86 ± 0.43 mm and 5.48 ± 2.06°, respectively. No statistical difference was noted between pre-operative planning and post-surgical analysis (*p* < 0.001). For animal trials, 12 New Zealand rabbits were recruited and divided into six pairs of AR and surgical guide groups. The animals were scanned again for error analysis in order compare deviation between AR group and surgical guide group. For bilateral condylar outer poles, distance deviations for AR and surgical guide groups were 0.93 ± 0.63 mm and 0.81 ± 0.30 mm, respectively (*p* = 0.68). For bilateral mandibular posterior angle, they were 2.01 ± 2.49 mm and 2.89 ± 1.83 mm, respectively (*p* = 0.50). Whereas, for mandibular osteotomy surface, distance deviation for both groups were 1.41 ± 0.61 mm and 1.21 ± 0.18 mm, respectively (*p* = 0.45) and the angular deviation were 6.81 ± 2.21° and 6.11 ± 2.93°, respectively (*p* = 0.65). The distance deviation for reconstructed tibiofibular osteotomy surfaces were 0.88 ± 0.22 mm and 0.84 ± 0.18 mm (*p* = 0.70), whereas, angular deviations were 6.47 ± 3.03° and 6.90 ± 4.01° (*p* = 0.84), respectively. There was no statistical difference between two groups (*p* > 0.05). In conclusion, AR system is feasible with accuracy similar to surgical guide. It also enhances surgeon’s hand-eye coordination during surgeries. However, more testing is required prior to clinical implementation
Prasad et al. (2023)	Prasad BA et al., 2023	Aim: This article demonstrated the feasibility and accuracy of AR-guided re-resections of head, neck and oral cavity cancers. 20 different dissections were performed on 3 cadavers by 2 head and neck surgeons. Before simulating cancer resection, two sutures were placed: one at the edge of the resection site(S) and other at adjacent resection bed(R). After resection, 2 fiducial markers were used to pinpoint the precise location of stitch R, which was then removed. Using a HMD, surgeons manually align the AR hologram to resection bed. Stitch S on the hologram was used as a guide to relocate stitch R and position a new stitch R′. The distance between R and R′ was measured for accuracy Results/Conclusion: The statistical analysis showed a mean relocation error of 4.0 ± 3.9 mm. However, errors pertaining to maxillary and mandibular resections differed significantly from those associated with other resections (10.7 vs. 2.8 mm; *p* < 0.01). After evaluating its applicability on cadavers, the authors emphasized on applying this technology to patients in the operating room
Gao et al. (2022)	Gao et al., 2022	Aim: Authors conducted a study aimed at treatment of craniofacial fibrous dysplasia with AR navigation and analyze its usability in cranio-maxillofacial surgery. Randomly selected data from five patients with craniofacial fibrous dysplasia was utilized for virtual planning and 3D printing. As a reference for estimating the bony resection, the normal contralateral side of the cranium was mirrored on the diseased side. The virtual recontouring plan was then superimposed on a 3D skull model. For AR registration, a marker fixed to patient’s dental model is tracked by HoloLens and Optical Tracking System (OTS), which was also used to track the surgical drill in real time. For accuracy measurement, a post-operative 3D model was superimposed onto a pre-operative surgical plan. Using software for 3D analysis, discrepancies were measured Results/Conclusion: The mean error across all five recontoured cranium models using AR guidance was 1.036 ± 0.081 mm. It was concluded that the AR treatment modality for craniofacial fibrous dysplasia is both effective and safe
Kim et al. (2020)	Jin Kim et al., 2020	Aim: In this case report, virtual planning and AR guidance were employed for the resection of a fibrous histiocytoma involving maxillary sinuses on left side Clinical symptoms: A 39-year-old Korean presented with facial deformity and discomfort in the left maxillary and palate region Investigations: Physical examination revealed no cervical lymphadenopathy and imaging showed radiolucent lesion. A CT scan revealed an expansile mass obstructing the left maxillary sinus entirely and extending into the nasal cavity Pre-requisites: Four osteotomies were planned for the total maxillectomy according to pre-surgical virtual planning. The AR viewer could track forehead marker and overlay images on actual patient. Additionally, a patient-specific polycaprolactone mesh implant was 3D printed for reconstruction of the orbital floor. For intervention, a Modified Weber Ferguson incision was made and AR guided surgery was performed using AR viewer. Osteotomies were performed and post-operative CT scan was collected to assess discrepancies between the pre-planned osteotomies and the actual osteotomies performed Results/Conclusion: The discrepancies in frontomaxillary, pterygomaxillary, pre-maxillary and zygomaticomaxillary osteotomies were 4.99, 2.25, 1.95, and 1.88 mm, respectively, with overall mean of 2.77 ± 1.29 mm. In conclusion, total maxillectomy performed under the AR guidance is appeared to be a powerful tool in applied surgery
Yang et al. (2022)	Yang et al., 2022	Aim: The authors examined the effect of cross-linking a mixed reality display device (HoloLens) and an optical navigator, analysing its accuracy with enhanced spatial relationship and graphical conversion on four patients with mandibular tumor. Imaging data was gathered for pre-operative 3D reconstruction and planning, and it was compared with 1 month postoperative data via error distribution map (3DMeshMetric) for error analysis Results/Conclusion: Using six marker points (anatomical landmarks), point registration was performed with a controlled registration error of less than 1 mm. In all cases, the surgical error analysis revealed a maximum error of 6.08 mm, with 4.79 mm in the majority of areas. Due to variations in chin surface and fibular morphology for reconstruction, the error is confined to the area of the chin in all cases. However, the authors highlighted that despite the fact this system enhances spatial experience and work efficiency, the overall accuracy still does not meet clinical requirements
Tang et al. (2022)	Tang et al., 2022	Aim: The authors conducted a retrospective study on seven patients (four with a maxillary tumor and three with a mandibular tumor) to evaluate the efficacy and accuracy of mixed reality augmented by surgical navigation. Virtual surgical plan with osteotomy planes was designed following image acquisition and fusion. Moreover, integration of mixed reality and surgical navigator was accomplished through IGT-link port for the transmission of image data between workstations. Using a HMD (HoloLens), osteotomy lines were distinguished by predetermined reference points using a navigation probe, and the spatial relationships between the probe and approaching structures were displayed on the user’s retina Results/Conclusion: The target error was set at 2 mm and intra-operative frozen section biopsies were performed to guarantee negative resection. A CT scan was ordered 1 week after surgery. Both pre-operative and post-operative 3D models were analyzed for accuracy. In total of 13 groups of osteotomy planes, the mean deviation from pre-defined osteotomy plane was 1.68 ± 0.92 mm and 80.16% of mean deviations were within 3 mm. Whereas, in context of maxillary and mandibular tumor, the mean deviations were 1.60 ± 0.93 mm and 1.86 ± 0.93 mm, respectively. During the follow-up period, no significant complications or recurrences were observed in any patients. Although considered safe and effective during oral and maxillofacial surgeries, the authors emphasized the need for additional research on surgical navigation and mixed reality application
Scolozzi and Bijlenga (2017)	Scolozzi et al., 2017	Aim: In this case report, researchers utilized tracked-microscope-based AR system for excision of pleomorphic adenoma of lacrimal gland Clinical Symptoms: In 2015, a 42 years old female was presented with facial asymmetry and an asymptomatic, slow growing mass in the upper lid of her left eye, resulting in restricted movement and diminished visual acuity Investigations: CT imaging showed multilobulated mass in the left lacrimal gland as well as erosion of upper and lateral orbital walls. Later, biopsy confirmed pleomorphic adenoma Results/Conclusion: The en-bloc excision was performed using AR and neuronavigation. The authors emphasized that this system was beneficial for identification, extension of lesion as well as en-bloc resection. At 2 years imaging follow-up, no recurrence was detected
Cercenelli et al. (2022)	Cercenelli et al., 2022	Aim: Authors evaluated the achievable registration accuracy and the success rate in performing an AR-guided skin paddle incision in fibular flap harvesting for mandibular reconstruction. They performed the experiment on a patient-specific 3D printed phantom and two display solutions (tablet and HoloLens 2) were compared Results/Conclusion: On average, the marker-less AR protocol showed comparable registration errors (ranging within 1–5 mm) for tablet-based and HoloLens-based solution. In 97% and 100% of cases, the AR-guided task was performed with an accuracy of ±2 mm (error margin of 4 mm), for tablet and HoloLens, respectively. The authors concluded that the proposed marker-less AR protocol can be suitable for assisting skin paddle harvesting in clinical setting
Scherl et al. (2021)	Scherl et al., 2021	Aim: Authors reported the first ever trial of AR-assisted surgery for parotid tumor ablation on six patients. Segmentation was done using MRI images and 3D hologram is manually overlaid on patient’s skin to estimate the position of tumor border. Alignment accuracy was measured through pre-defined landmarks using electromagnetic navigation device Results/Conclusion: The mean error of alignment was 1.3 cm (0.5–2.1). Statistical difference was noted among central and peripheral structures showing better accuracy centrally (*p* = 0.0059). No long-term complication was noted in any AR-guided case. However, authors emphasized that further work need to be done on skin surface registration and alignment to improve accuracy
Necker et al. (2023)	Necker et al., 2022	Aim: Authors introduced a technique for flap sizing after mandible tumor resection on cadaver. A resected mandibular tumor was 3D scanned using smartphone and annotated with colours for orientation. The 3D scanned virtual specimen was displayed through AR-HMD Results/Conclusion: The 3D hologram was manually placed back in its original site accurately. After adjusting the size, the hologram was then overlaid on flap harvesting site for reconstruction planning. Authors concluded that this technique could assist in fibular flap reconstruction of mandibular tumor
Han et al. (2022)	Lin et al., 2022	Aim: Authors proposed an integrated approach in mandibular reconstruction based on 3 technologies: 3D printing, mixed reality (MR), Robotic-Assisted navigation (RAN). MR was used to output the visualized project and matched the anatomical 3D reconstruction model in reality. The 3D plate was printed for surgical guidance. RAN was used to guide and position the vascularized fibula autograft and the immediate dental implantation Results/Conclusion: Authors concluded that constructed MR, 3D, and RAN technologies assist each other to make the surgery more accurate and minimally invasive
Zhao et al. (2023)	Zhao et al., 2022	Aim: Authors reported a cadaver study to investigate a novel method of fibula free flap (FFF) osteotomy based on AR technology. AR-based surgical navigation was used to guide the FFF osteotomy and these fibular segments were used to reconstruct a defective mandible model. After reconstruction, all segments were scanned by CT and osteotomy accuracy was evaluated by measuring the length and angular deviation between the virtual plan and the final result, and the volume overlap rate and average surface distance between the planned and obtained reconstruction Results/Conclusion: The length difference, angular deviation, volume overlap rate and average surface distance were 1.18 ± 0.84 mm, 5.45 ± 1.47°, 95.31% ± 2.09%, and 1.22 ± 0.12 mm, respectively
Modabber et al. (2022)	Modabber et al., 2021	Aim: Authors proposed AR guided solution for harvesting grafts for mandibular reconstruction and demonstrated its clinical application and surgical accuracy. They used human cadavers to demonstrate a projector-based marker-less AR setup for harvesting iliac crest grafts and compared it to a cutting guides procedure. A total of 10 iliac crests from 5 cadavers were used, and each iliac crest was randomly assigned to either harvested using AR guidance or using cutting guides. The transplants were digitized using CBCT and accuracy was measured in terms of angles, distances, and volumes of the actual and intended osteotomies Results/Conclusion: Both AR and cutting guides effectively replicated the virtually projected transplant volume with precision. Nevertheless, there was a significant difference (*p* = 0.018) in the cumulative angulation of the osteotomy plane between the AR group and the group using cutting guides (14.99 ± 11.69° vs. 8.49 ± 5.42°). The results indicate that the accuracy of AR-guided navigation in terms of cumulative osteotomy plane distance was lower (2.65 ± 3.32 mm) compared to the cutting guides (1.47 ± 1.36 mm), however this difference was not statistically significant. Furthermore, more research was recommended before clinical implementation
Meng et al. (2021)	Meng et al., 2021	Aim: Authors reported a cadaver study to investigate the feasibility of the application of MR in mandible reconstruction with fibula flap. AR was used to guide the osteotomy and shaping of the fibular bone. After fixing the fibular segments using the titanium plate, all segments underwent a CT examination. The planned and the actual postoperative fibula osteotomies were compared in terms of angular deviation of fibular segments, and intergonial angle distances. To evaluate the accuracy of MR technique, the distance between the postoperative actual cutting edge and preoperative osteotomy plan of each fibular segment’s was calculated Results/Conclusion: The mean location of the fibular osteotomies, angular deviation of the fibular segments, and intergonial angle distances were 2.11 ± 1.31 mm, 2.85° ± 1.97°, and 7.24 ± 3.42 mm, respectively
Battaglia et al. (2019)	Battaglia et al., 2019	Aim: Authors reported a case series of 3 consecutive patients who underwent mandibular reconstruction using AR-assisted fibular free flap harvesting. Using an app installed on a tablet, the virtual-to-real registration is performed according to a shape recognition system of the leg of the patient, rendering in real time a superimposition of the anatomy of the bony, vascular, and skin of the patient and also the surgical planning of the reconstruction Results/Conclusion: Accuracy of AR overlay was verified visually by the surgeons, who believe that AR can be a prospective improving technology for mandibular complex reconstruction
Winnand et al. (2022)	Winnand et al., 2021	Aim: The objective of this research is to examine the usability, visual perception, and accuracy of projector-based marker-less navigation of AR guided harvesting of the iliac crest, in comparison to harvesting using cutting guides for facial abnormalities on phantoms. A random selection of 5^−ΔΔCT^ scans was made to enroll a total of 10 iliac crests. Two commonly shaped transplants (box shaped and hockey stick shaped) were virtually planned. Each of the 10 iliac crests was replicated (3D printed) four times for the purpose of testing. Following the surgical removal, the transplants underwent digitization using CBCT. The accuracy of the measurements was assessed by quantifying the volume, distance, and angular deviation Results/Conclusion: In the context of volume rendering, the osteotomies supported by AR demonstrated higher levels of accuracy compared to those performed using a cutting guide (*p* = 0.057). Therefore, a statistically significant difference was seen in the accuracy of osteotomies for box-shaped transplants, but no significant difference was observed for hockey stick-shaped transplants. In general, the average discrepancy in osteotomy plane angulation from the virtual design was found to be 10.21 ± 7.22° with the assistance of AR, and 6.98 ± 4.70° when utilizing cutting guides. A notable disparity between the methods was observed, with a statistically significant difference (*p* = 0.004). Similarly, the mean differences between the osteotomy planes and the planned trajectories were determined to be 2.29 ± 1.98 mm for AR and 1.32 ± 1.00 mm for cutting guides. This analysis revealed a significant difference between the two methods (*p* = 0.002). Therefore, due to certain limitations in AR-guided harvesting further work on research was recommended

**FIGURE 2:**
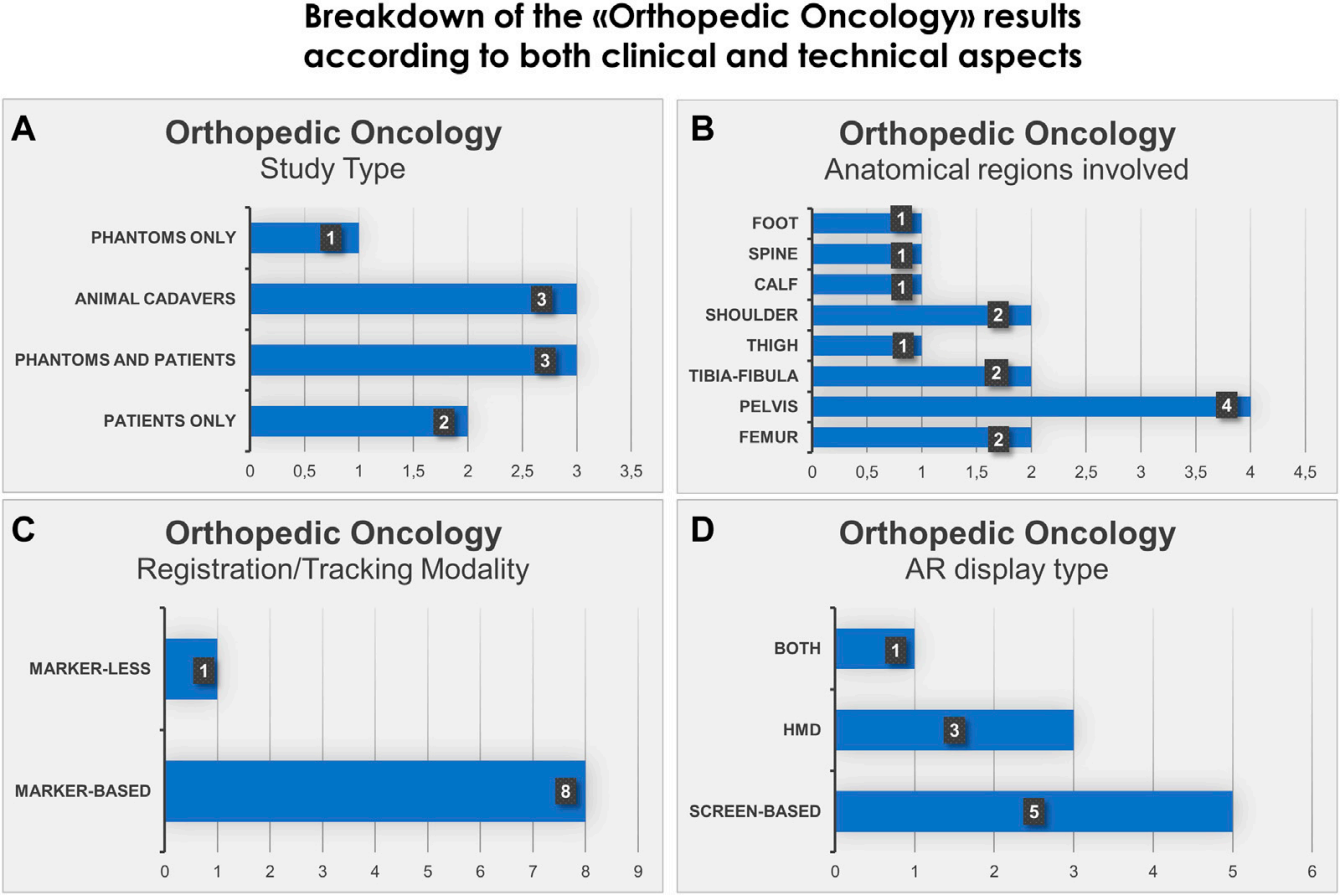
Bar histograms depicting types of study **(A)**, anatomical regions involved in the surgery **(B)**, registration/tracking modality **(C)** and AR display type **(D)**, for Orthopedic Oncology.

**FIGURE 3:**
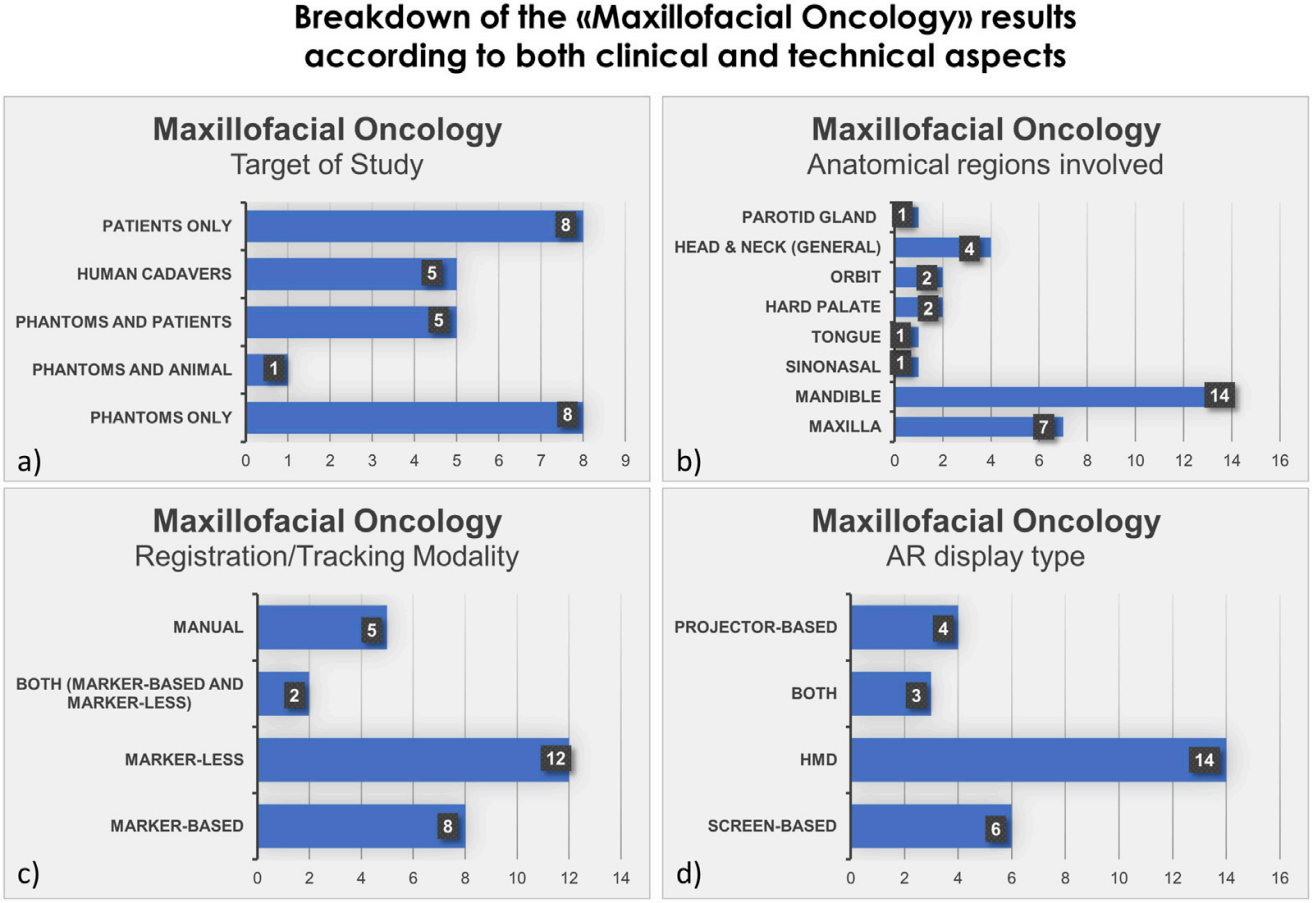
Bar histograms depicting types of study **(A)**, anatomical regions involved in the surgery **(B)**, registration/tracking modality **(C)** and AR display type **(D)**, for Maxillofacial Oncology.

We also show in bar histograms the breakdown of the studies according to some of the most interesting aspects above mentioned (Figures 2, 3).

### 3.1 Orthopedic oncology

For orthopedic oncology (Cho et al., 2017; Choi et al., 2017; Cho et al., 2018; Moreta-Martinez et al., 2018; Abdel Al et al., 2020; García-Sevilla et al., 2021a; Molina et al., 2021c; Moreta-Martinez et al., 2021; Pose-Díez-de-la-Lastra et al., 2022), four studies were conducted on phantoms (*n* = 1) or both on phantoms and prospectively on patients (*n* = 3). Three studies were conducted on animal cadavers, whereas, two studies directly employed AR on patients in OR. In the context of AR display and registration/tracking modality, five studies used screen-based display and eight studies opted for a marker-based registration/tracking modality. Regarding the anatomical region of interest, pelvis is the most involved anatomical region in the selected studies (*n* = 4).

The main outcomes reported in the included articles referred to the registration error of the AR systems, the AR-guided surgical accuracy in performing tumor resection compared to preoperative planning and/or to standard procedures (i.e., manual measurements), the placement error in positioning surgical guides or patient-specific implant under AR assistance.

Despite the fact that the application of AR in the field of orthopedic oncology has been relatively limited compared to maxillofacial oncology to date, the included studies demonstrate the potential future significance of AR technology in this surgical field.

### 3.2 Maxillofacial oncology

In the field of maxillofacial oncology (Scolozzi and Bijlenga, 2017; Battaglia et al., 2019; Gsaxner et al., 2019; Pepe et al., 2019; Kim et al., 2020; García-Sevilla et al., 2021b; Gsaxner et al., 2021; Meng et al., 2021; Ochandiano et al., 2021; Sahovaler et al., 2021; Scherl et al., 2021; Sugahara et al., 2021; Tel et al., 2021; Ceccariglia et al., 2022; Cercenelli et al., 2022; Chan H et al., 2022; Gao et al., 2022; Han et al., 2022; Modabber et al., 2022; Shi et al., 2022; Tang et al., 2022; Winnand et al., 2022; Yang et al., 2022; Necker et al., 2023; Prasad et al., 2023; Shaofeng et al., 2023; Zhao et al., 2023), in the majority of articles (*n* = 14) the proposed AR systems were tested on phantoms, and 8 on patients only. Five researcher groups carried out pre-clinical research on phantoms before using AR on patients. The marker-less registration approach (*n* = 14) and wearable AR displays, i.e., HMDs (*n* = 14), were utilized in the majority of the research studies. These two investigating factors made up 52% of the total studies. Mandible, being the most involved anatomical area of interest for AR implementation, comprises of 52% of total studied areas (*n* = 14).

The following are the primary findings that are covered in the articles: the registration error, the AR-guided surgical accuracy in performing tumor resection or flap harvesting compared to preoperative planning, and the AR-guided surgical accuracy compared to the more conventional use of 3D printed cutting guides. It is interesting to mention that, some studies in maxillofacial oncology have also incorporated external tracking navigation systems into augmented reality to improve accuracy and spatial relationships. The articles included have shown the importance of AR and its future perspectives in this field. Nevertheless, the majority of the studies underlined the need for additional research before clinical application.

## 4 Discussion

In the set of orthopedic oncology articles, all studies utilized marker for registration except 1 (Abdel Al et al., 2020), which opted for marker-less registration. Mean registration error found to be less than 3 mm where measured (not all articles reported the registration error) excepting the one case of complex shoulder phantom (Pose-Díez-de-la-Lastra et al., 2022), where RMSE was slightly more than 3 mm with HoloLens 2.

Out of nine articles, only three randomized controlled trial studies (RCT) were reported (performed on animal cadavers). Mean AR-assisted resection was less than 2 mm as compared to conventional approach (e.g., manual resection) which had mean resection error more than 2.5 mm in these studies (Cho et al., 2017; Choi et al., 2017; Cho et al., 2018).

Included studies of orthopedic oncology also reported two cases, both achieved intended outcomes using AR without any complications (Abdel Al et al., 2020; Molina et al., 2021c). Moreover, one study demonstrated a better positioning of surgical guide in simulated pelvic tumor through AR as compared to freehand method (García-Sevilla et al., 2021a).

Regarding usability, only one study conducted a questionnaire survey with patients and surgeons, and the results turned out to be satisfactory (Moreta-Martinez et al., 2021). Ergonomics of the most popular AR devices, HoloLens 1 and 2, were discussed by Pose-Díez-de-la-Lastra et al. (2022) while comparing them on two orthopedic cases. They indicated that HoloLens 2 has superior ergonomics (score 4 out of 5) as compared to HoloLens 1 (score 2.84 out of 5).

The primary outcomes of the included articles in orthopedic segment demonstrated the utility of AR and showed even better results than conventional procedures in some cases. However, some of the limitations of the orthopedic segment is that few articles discuss the usability and ergonomics of this technology. Only three out of nine articles are randomized controlled trials (RCTs), making it difficult to derive a statistical comparison value from the current studies. Furthermore, only four articles provided surgical accuracy (Cho et al., 2017; Choi et al., 2017; Cho et al., 2018; García-Sevilla et al., 2021a) and only four provided registration error (Choi et al., 2017; Moreta-Martinez et al., 2018; Moreta-Martinez et al., 2021; Pose-Díez-de-la-Lastra et al., 2022) showing the limitation of this research.

Over all, with consideration of sufficient accuracy achieved in terms of registration error and surgical accuracy during surgical simulations, it can be said that AR is beneficial and helpful in orthopedic oncological surgeries. However, due to limited literature in orthopedic oncology till date, further testing is recommended.

In maxillofacial oncology, out of eight marker-based studies, only two reported AR regisration error in terms of fiducial registration error (<1 mm) (Sahovaler et al., 2021; Chan H et al., 2022). Marker-less registration error (where measured in articles) ranges from less than 1 mm to upto 2 cm (Gsaxner et al., 2019; Gsaxner et al., 2021; Cercenelli et al., 2022; Shi et al., 2022; Yang et al., 2022). This shows that some articles did not achieve the desired precision in AR registration through the marker-less approach.

Marker-based AR surgical accuracy was reported in five articles and it was demonstrated in terms of deviation from pre-planned surgical resection. Mean surgical resection error was measured to be less than 3 mm among these articles (Kim et al., 2020; García-Sevilla et al., 2021b; Chan H et al., 2022; Gao et al., 2022; Zhao et al., 2023). Conversely, seven articles reported surgical accuracy using the marker-less approach. The accuracy ranges from 0.49 to 2.77 mm in six articles (Ceccariglia et al., 2022; Cercenelli et al., 2022; Modabber et al., 2022; Shi et al., 2022; Tang et al., 2022; Winnand et al., 2022), whereas, one study had a maximum surgical error of 6.08 mm which did not meet surgical requirement (Yang et al., 2022).

Two studies employed both marker-less and marker-based registration, the latter being used mainly in the case of lack of surface features easily recognizable (Shaofeng et al., 2023) or to introduce a calibration procedure aimed at improving registration accuracy (Pepe et al., 2019). Particularly in (Shaofeng et al., 2023) the surgical accuracy, in terms of distance deviation between the planned osteotomies and postoperative cuts performed under AR guidance, was measured in the range of 0.88–2.01 mm. Manual alignment for virtual-to-real registration was performed in five articles (Meng et al., 2021; Scherl et al., 2021; Han et al., 2022; Necker et al., 2023; Prasad et al., 2023) and only two of them reported errors in terms of overlaying AR images. One study showed average relocation error of 4 mm ± 3.9 mm with HoloLens 2 (Prasad et al., 2023), whereas the other measured mean registration error of 1.3 cm with HoloLens 1 (Scherl et al., 2021). Even though it is hard to comment based on only two results, it seems that chances of error are more in manual registration as compared to marker-less or marker-based approach.

Two of the three case reports on maxillofacial oncological surgery were qualitative, while one case of fibrous histiocytoma had quantified findings with an overall mean discrepancy of 2.77 ± 1.29 mm using AR (Kim et al., 2020). According to qualitative studies, the incorporation of mixed reality during pre-surgical and intra-operative phases allows for precise surgical outcomes and is helpful for lesion identification and determination of its extension (Scolozzi and Bijlenga, 2017; Sugahara et al., 2021).

A total of seven studies have examined the utilization and precision of AR in the context of flap harvesting for mandibular restoration (Battaglia et al., 2019; Meng et al., 2021; Cercenelli et al., 2022; Han et al., 2022; Modabber et al., 2022; Winnand et al., 2022; Zhao et al., 2023). Among these, two of them conducted a comparison between marker-less AR guidance and cutting guides in the context of iliac crest harvesting (Modabber et al., 2022; Winnand et al., 2022). The results indicated that cutting guides exhibited superior precision compared to AR navigation in terms of, both distance and angular deviation from pre-determined trajectories. However, the distance deviations were less than 2.7 mm in AR group. In contrast, Zhao et al. (2023) employed a marker-based methodology to assess the fibular flap, yielding an average distance deviation of 1.22 ± 0.12 mm. One possible explanation for this phenomenon is that the marker-based approach tends to exhibit more accuracy in comparison to the marker-less approach in relation to lower limb bones. This might be attributed to the specific shape and contour of these bones, which pose challenges for marker-less registration techniques.

It is interesting to outline that five of the studies utilized external navigator system with mixed reality. Four of them incorporated external navigation into mixed reality to enhance accuracy and spatial relationships (Scolozzi and Bijlenga, 2017; Gao et al., 2022; Tang et al., 2022; Yang et al., 2022) and one study compared AR and optical tracking system (OTS) accuracy (García-Sevilla et al., 2021b).

García-Sevilla et al. (2021b) compared AR and OTS for surgical navigation and concluded that they have similar accuracy with errors below 1 mm. Gao et al. (2022) used HoloLens and OTS on five patients of cranio-fibrous dysplasia and the mean registration error across all cranium models was 1.036 ± 0.081 mm. Tang et al. (2022) conducted a study to evaluate efficacy and accuracy of mixed reality, which is augmented by surgical navigator, on seven patients of maxilla and mandibular tumors. The mean deviation from pre-defined osteotomy plane was 1.68 ± 0.92 mm (set target error was 2 mm). However, in the study by Yang et al. (2022), cross linking of mixed reality and optical navigator did not produce clinically required accuracy (maximum error was 6.08 mm), but authors highlighted that this system enhanced spatial experience and work efficiency. Furthermore, in one case report, researchers utilized tracked-microscope-based AR system and they emphasized that this system was helpful in identifying and determining the extension of pleomorphic adenoma of lacrimal gland (Scolozzi and Bijlenga, 2017).

Feasibility and usability studies were conducted in two articles in maxillofacial oncology. In one study, the standard ISO-9241/110 feasibility questionnaire based on the 5-point Likert Scale was conducted on both medical staff and AR experts, and overall feedback turned out to be positive (Pepe et al., 2019). In another article by Gsaxner et al. (2021), AR usability evaluation received a SUS of 74.8 ±15.9 (>68 indicates above average) with the 5-point Likert scale of 4.5 ± 0.7 out of 5 (5 representing extremely positive).

In addition, AR guided simulations scored higher as compared to virtual unguided resections in mental demand, performance, effort and frustration in preclinical study conducted on phantoms by Chan H et al. (2022).

Some clinicians find the AR system simple to learn and use, which improves their decision-making skills (Gsaxner et al., 2021), whereas, some authors stressed on the importance of pre-clinical education in managing the technology’s steep learning curve (Ochandiano et al., 2021).

Despite of the fact that a few studies did not achieve sufficient accuracy in terms of registration error and surgical accuracy in maxillofacial oncology, the overall outcomes seem to have a positive impact of AR in maxillofacial oncological surgeries. For instance, according to Chan H et al. (2022), AR guided resection improved negative margin and had more similarities with pre-planned cutting planes. Similarly, Shaofeng et al. (2023) came to conclusion that AR system accuracy is similar to that of surgical guide while testing it on mandibular tumor and fibular reconstruction. They also emphasized that it enhances surgeon’s hand-eye coordination in executing surgeries. Ceccariglia et al. (2022) found the discrepancy of under 2 mm between AR projected osteotomy and customized cutting guide osteotomy. Shi et al. (2022) concluded that AR navigation can effectively display and guide the surgical path and helps in achieving desired results. Furthermore, Sahovaler et al. (2021) showed advantage of AR over unguided simulations. However, some pressing concerns like limited depth perception and time required for auto-registration were also mentioned in some studies (Pepe et al., 2019; Gsaxner et al., 2021; Modabber et al., 2022).

Similar to orthopedic oncology, maxillofacial oncology section also lacks in many aspects. Feasibility questionnaire survey and ergonomics are discussed in only few articles. The different methods of evaluation in articles limited the ability to provide quantifiable results. Additionally, only 10 out of 27 articles reported on registration error and only 14 reported on surgical accuracy. Conversely, further research is emphasized pre-clinically before implementing AR in operating rooms.

We should advocate for the development of a technique that is uniform and consistent in order to investigate this new technology and make it possible to conduct meta-analyses for future investigations. This stage is crucial for gathering data to support the use of AR in oncological procedures in both disciplines. Additionally, we stress the importance of including external navigation in AR in future experiments in order to enhance the precision and depth perception of this infant, yet useful technology. Moreover, through the use of standardized questionnaires, SUS, and a 5-point Likert scale, feasibility and ergonomics should be evaluated.

We observed from the collected papers that an important aspect of AR implementation is three-dimensional printing (3D printing), also referred to as rapid prototyping, which is typically used for obtaining pre-operative patient-specific phantoms replicating the anatomical structures of interest. These phantoms are typically used in the papers to perform the surgical task preoperatively under AR guidance, as well as to evaluate both the registration error and surgical accuracy (Moreta-Martinez et al., 2018; Gsaxner et al., 2019; Pepe et al., 2019; García-Sevilla et al., 2021a; García-Sevilla et al., 2021b; Gsaxner et al., 2021; Moreta-Martinez et al., 2021; Ochandiano et al., 2021; Sahovaler et al., 2021; Tel et al., 2021; Chan H et al., 2022; Gao et al., 2022; Pose-Díez-de-la-Lastra et al., 2022; Shi et al., 2022; Shaofeng et al., 2023). When using a marker-based tracking approach, the reference marker which is designed to fit in a unique position on the patient, is produced by 3D printing (Moreta-Martinez et al., 2021; Cho et al., 2017; Choi et al., 2017; Cho et al., 2018; Moreta-Martinez et al., 2018; García-Sevilla et al., 2021b; Ochandiano et al., 2021; Sugahara et al., 2021; Pose-Díez-de-la-Lastra et al., 2022; Shaofeng et al., 2023). Moreover, in some cases (Moreta-Martinez et al., 2018; Ceccariglia et al., 2022) patient-specific surgical guides used for comparative evaluation with AR guidance on surgical accuracy are manufactured via 3D printing. Finally, some studies clearly suggest to use AR and 3D printing in combination to improve surgical efficacy, accuracy, and patients experience (García-Sevilla et al., 2021a; Moreta-Martinez et al., 2021).

### 4.1 Limitations of AR in surgery

Despite the fact that AR is a growing technology, it is not without limitations and complications. Surgeons should be well aware of the limitations of augmented reality in surgery, including technical challenges, limited field of view which limits the amount of virtual content available to the user, high implementation costs and limited user experience. In addition, they should consider how these limitations may impact the accuracy and efficacy of AR systems, as well as the surgical outcomes. Before incorporating AR into clinical practice, it is essential to execute a comprehensive analysis of its viability and benefits.

For instance, the viewing distance and angle of commercially available HMDs, such as HoloLens 2, are not optimized for use in surgery since the focus distance is suboptimal for medical procedures that are typically carried out at arm’s length and with the head bowed to observe the operative field (Wong et al., 2022).

To the authors’ knowledge, today only two “surgery-specific” headsets are available for AR-based intraoperative guidance: the X-vision Spine System by Augmedics, which received FDA approval (https://www.augmedics.com/), and the VOSTARS system, still under investigation (https://www.vostars.eu/). VOSTARS is promising new wearable AR system designed as a hybrid Optical-See-Through (OST)/Video-See-Through (VST) HMD capable to offer a highly advanced navigation tool for maxillofacial surgery and other open surgeries. An early prototype of the VOSTARS system (Ruggiero et al., 2023) has been already evaluated in phantom tests and demonstrated a sub-millimetric accuracy (0.5÷1 mm) in the execution of high-precision maxillofacial tasks (Cercenelli et al., 2020; Condino et al., 2020).

The issue of depth perception is another challenge that surgeons have to consider when applying AR technology during surgical procedures (Sielhorst et al., 2006). Surgeons must accurately gauge the distance between their instruments and the intended targets for AR surgery to be successful. However, accurate distance estimation during AR-assisted surgery is complicated by the fact that tools and target landmarks are 3D-rendered (Choi et al., 2016).

In order to implement AR in surgery, complex technical solutions, including medical-grade software and hardware systems, are required. For example, consumer-grade computer systems are suboptimal for displaying high-quality 3D rendered objects, and HMDs have a limited battery life (2–3 h), which can result in technical issues such as system failure, calibration errors, and latency. These obstacles may limit the accuracy of AR systems and result in surgical complications (Wong et al., 2022).

In addition, surgeons using AR-HMDs must contend with the limited field of view, restricted binocular field and projection size (Lareyre et al., 2021).

Virtual-to-real scene registration with HMDs is another major issue when using AR for surgical guidance and simulation, resulting in inaccurate identification of the deep anatomical structure in question. Due to the fact that these display devices were not devised for medical purposes, their technical characteristics are less suited for surgical procedures (Badiali et al., 2020).

Surely, a marker-less registration, i.e., without the use of fiducial markers or trackers anchored to the patient, is highly preferable in surgery, however it is not always feasible for certain surgeries. As also emerged from our analysis, in maxillofacial oncological surgery a marker-less based approach seems to be more viable since the edges of anatomical parts (e.g., mandible or skull) are more accessible and trackable during surgery; conversely, in orthopedic field the intraoperative recognition of bone edges may be more difficult.

During surgery, a surgeon using an AR headset may endure discomfort, weariness, eye strains, and headache. Furthermore, it is possible that the surgeon’s ability to focus on the surgical operation field might get impaired due to the visualization of augmented information. However, in a study conducted on simulation sickness, in 2018, authors showed that out of 142 HMD’s users from various fields, only few experienced mild discomfort (Vovk et al., 2018).

Because this technology is high priced and requires significant investment to initiate and implement in hospitals, it is not widely accessible in all medical settings. Consequently, the data associated with augmented reality in the surgical field are rather preliminary and require further testing and analysis.

The AR application has a steep and costly learning curve. It requires expertise, and most surgeons are unfamiliar with AR utilization. So they must collaborate with biomedical engineers to implement this technology in the operating room. Personnel must endure time-consuming hands-on training in order to implement AR in hospital surgical setups.

When information needs to be electronically distributed to several departments in order to make patient-specific tools for AR, patient data privacy is another concern that must be addressed. When dealing with sensitive information pertaining to patients, certain national regulations should be followed in order to protect and secure both their safety and their privacy.

### 4.2 Future of AR

Augmented reality is being recognized as a promising application for enhancing the outcomes and standard of care for orthopedic and maxillofacial oncology patients. Especially in complex oncological cases that require a strategic planning for execution and comprehension, it is emphasized that surgeons should consider using augmented reality where applicable, in combination with 3D printing. However, implementation of AR and its tools in surgical cases and healthcare has certain shortcomings which can be improved with future advancement in technology. Depending on the complexity of cases, process from procuring CT scan/MRI images to 3D printing of pre-surgical phantoms and developing a patient-specific AR application take days, sometimes even months including preoperative surgical planning and simulation training. Surgeons and biomedical engineers must work together to refine and successfully execute the procedure. Therefore, as future perspective, AR cannot be used in emergency cases and can only be advised in elective surgeries. Despite these facts, we continue to believe that AR technology has the potential to revolutionize the conventional methods of oncological surgical procedures and overcome all of these limitations. Augmented reality is expected to help in better understanding of tumor anatomy and in plan the resection accordingly. This will contribute to improve the outcomes and standard of care by limiting the recurrence rate while attaining the desired surgical margins with accuracy.

## 5 Conclusion

Currently, augmented reality is one of the most innovative technology in the field of surgery, particularly orthopedic and maxillofacial surgery. Due to its real-time visualization of preoperative images and planning directly on the patient, AR can be particularly beneficial for oncological surgeries in both fields to achieve the desired surgical accuracy. In oncological surgery, the AR allows to overcome some limitations of conventional computer-assisted surgical navigation, such as the surgeon’s attention shift from the operative field to view the navigation monitor, as well as to avoid the lead-time in manufacturing 3D-printed cutting guides. Indeed, AR should be used as a complementary tool to other computer-assisted technologies, as suggested by our literature review: particularly for maxillofacial oncology, surgeons have begun to incorporate external navigation systems into AR to track the surgical probe or instruments, to further improve accuracy and spatial relationships.

Even though AR technology is still in its infancy and has certain limitations, the current outcomes of its application in both disciplines are promising to support its clinical use. Certain concerning aspects still remain, related to image-to-patient registration and surgical accuracy. In the present review, we attempted to identify a range of registration error and surgical accuracy based on results from both surgical domains. Although it is difficult to derive general ranges due to the involvement of various anatomical regions and different complexity for each domain it can be observed that AR resection error exhibited greater accuracy compared to conventional un-guided resection, hence successfully attaining the desired goals without any associated complications. Additionally, in some studies the AR navigation showed comparable accuracy with pre-planned virtual cutting planes and with customized cutting guides.

We believe that the still limiting technical aspects on registration and surgical accuracy can be improved and overcome with further development in hardware and software used for AR. For that, industry-academic partnerships are essential to advance the technology, in conjunction with clinical studies to assess its benefits and role in the clinical practice.

In conclusion, although AR is seen to have the capacity to enhance surgical efficiency and ensure patient’s safety, further search needs to be done pre-clinically in order to improve its accuracy and before achieving its wide adoption in clinical settings.

## Data Availability

The raw data supporting the conclusion of this article will be made available by the authors, without undue reservation.
